# Radiofrequency ablation vs. cryoablation for pediatric atrioventricular nodal reentrant tachycardia in the era of three-dimensional electroanatomical mapping

**DOI:** 10.3389/fcvm.2025.1527768

**Published:** 2025-01-30

**Authors:** Yao-Wei Chan, Chieh-Mao Chuang, Pi-Chang Lee, I-Hsin Tai, Ying-Hsuan Peng, Wen-Po Fan, Yu-Shin Lee, Ming-Chih Lin, Sheng-Ling Jan, Yun-Ching Fu, Shih-Ann Chen

**Affiliations:** ^1^ Division of Pediatric Cardiology, Children’s Medical Center, Taichung Veterans General Hospital, Taichung, Taiwan; ^2^ Division of Cardiology, Department of Internal Medicine, Asia University Hospital, Taichung, Taiwan; ^3^ Department of Cardiology, China Medical University Children’s Hospital, Taichung, Taiwan; ^4^ Department of Pediatrics, College of Medicine, China Medical University, Taichung, Taiwan; ^5^ Division of Pediatric Cardiology, Department of Pediatrics, Chung Shan Medical University Hospital, Taichung, Taiwan; ^6^ Division of Pediatric Cardiology, Department of Pediatrics, Taipei Veterans General Hospital, Taipei, Taiwan; ^7^ Division of Pediatric Cardiology, Department of Pediatrics, Chang Gung Memorial Hospital, Linkou Branch, Taoyuan, Taiwan; ^8^ Department of Post-Baccalaureate Medicine, College of Medicine, National Chung Hsing University, Taichung, Taiwan; ^9^ Cardiovascular Center, Taichung Veterans General Hospital, Taichung, Taiwan; ^10^ Institute of Clinical Medicine, and Cardiovascular Research Center, National Yang-Ming Chiao-Tung University, Taipei, Taiwan

**Keywords:** pediatric, atrioventricular nodal reentrant tachycardia, radiofrequency ablation, cryoablation, electroanatomical mapping systems

## Abstract

**Background:**

Cryoablation for pediatric atrioventricular nodal reentry tachycardia (AVNRT) is favored for reducing conduction system injury compared to radiofrequency (RF) ablation. The safety advantage of cryoablation over RF ablation primarily results from studies conducted without a three-dimensional electroanatomical mapping (3D EAM) system. Currently, 3D EAM systems offer precise and efficient guidance, improving safety and outcomes. This study compares RF ablation and cryoablation using a 3D EAM system for pediatric AVNRT treatment.

**Methods:**

A retrospective study enrolled consecutive pediatric patients with AVNRT who underwent RF ablation (RF group) or cryoablation (Cryo group) guided by a 3D EAM system at multiple centers from July 2018 to January 2024.

**Results:**

Among 95 patients, 69 received RF ablation and 26 received cryoablation. Recurrence rates were 2.9% in the RF group and 0% in the Cryo group (*p* > 0.99), with no difference in AVNRT-free survival. No major complications, such as permanent atrioventricular (AV) block, were observed. The minor complication rates, including transient AV block, did not differ significantly (14.5% vs. 11.5%, *p* > 0.99). The RF group had a significantly shorter procedure time (111 vs. 153.5 min, *p* = 0.005). Ablation outside the low Koch triangle and cryoablation were independently associated with longer procedure times. The procedure time decreased significantly in the recent 50% of RF ablation cases, but not in cryoablation cases.

**Conclusion:**

With 3D EAM guidance, both RF ablation and cryoablation are considered safe and effective for pediatric AVNRT. RF ablation is more efficient with a shorter procedure time after increasing experience.

## 1 Introduction

Atrioventricular nodal reentrant tachycardia (AVNRT) is a common type of supraventricular tachycardia (SVT), accounting for approximately one third of ablation substrates in a recent prospective multicenter registry study in pediatric population (1). Although AVNRT is generally considered to have a benign course, it can be associated with ventricular dysfunction, acute hemodynamic compromise, and the need for emergency medical care. Furthermore, medical therapy is sometimes ineffective or associated with intolerable adverse effects. Therefore, some patients or families tend to avoid chronic use of antiarrhythmic medications. Catheter ablation is currently widely used as a curative therapy (2).

The standard approach for catheter ablation of AVNRT involves modification or elimination of the slow pathway. There is controversy regarding the choice between radiofrequency (RF) ablation and cryoablation for these procedures. In recent years, cryoablation has emerged as the preferential energy choice for pediatric AVNRT due to its lower incidence of atrioventricular (AV) block (3–7).

To date, there is a lack of meta-analysis or randomized controlled trials comparing the two ablation energies in pediatric patients with AVNRT. The available meta-analyses involving adults and children indicate a higher incidence of AV block in the RF group (0.75%−0.87% vs. 0%) (8, 9). However, in recent pediatric data, a large retrospective cohort study involving 504 pediatric patients with AVNRT reported only one case that required permanent pacemaker implantation due to high-grade AV block (0.2%) (10).

The lower incidence of AV block in the modern era could potentially be attributed to the strength of 3D EAM, which has been associated with a reduced risk of complete heart block related to the procedure (11). We hypothesize that 3D EAM can counterbalance the risks associated with RF ablation while preserving its other advantages, such as a shorter procedure time, a lower recurrence rate, and a lower cost.

In addition, the use of 3D EAM enables the feasibility of non-fluoroscopic catheter ablation, further reducing the potential harm related to radiation exposure for both patients and medical personnel. Currently, there is a lack of studies comparing RF ablation and cryoablation guided by the 3D EAM system for the treatment of children with AVNRT. Our study aims to compare the feasibility, safety, and effectiveness of RF ablation and cryoablation with the guidance of a 3D EAM system for the treatment of pediatric AVNRT.

## 2 Methods

### 2.1 Study population

In this retrospective study, we analyzed 95 consecutive pediatric patients with AVNRT who underwent ablation procedures performed in two medical centers in Taiwan from July 2018 to January 2024. The inclusion criteria were symptomatic children aged 18 years or younger with AVNRT confirmed by electrophysiological study (EPS), who received RF ablation (RF group) or cryoablation (Cryo group) under the guidance of a 3D EAM mapping system. The demographic and clinical data collected included the patient's age, gender, body weight, body surface area, medical history, and clinical manifestations. We defined the young age group using the first quartile of the age distribution as a cut-off value. Physical examinations, laboratory examinations, electrocardiogram (ECG) and echocardiography were performed before the procedure. Each patient obtained informed consent, and the study protocol complied with the 1975 Helsinki Declaration's ethical guidelines and was prior approval by the Institutional Review Board of Taichung Veterans General Hospital (TCVGH-IRB No. CE22018A-2).

### 2.2 Pre-ablation procedures

Antiarrhythmic medications were discontinued for at least 5 half-lives prior to the procedures. Conscious sedation was administered according to the preferences and cooperability of the patients. EPS was carried out using a standardized atrial and ventricular stimulation protocol. AVNRT was diagnosed when there was evidence of dual AV nodal physiology and inducible tachycardia with typical electrophysiological features. Dual AV nodal physiology is defined as AH/VA jump greater than 50 ms when single extrastimulation by 10 ms decreases or sustained slow pathway conduction (SSPC), which is demonstrated by PR interval longer than RR interval during burst atrial pacing. If tachycardia could not be induced, isoproterenol was administered and EPS was repeated. All patients underwent procedures under the guidance of the 3D EAM system (Ensite NavXTM, Abbott, MN, USA), with or without fluoroscopy use.

### 2.3 RF ablation

**Figure 1:**
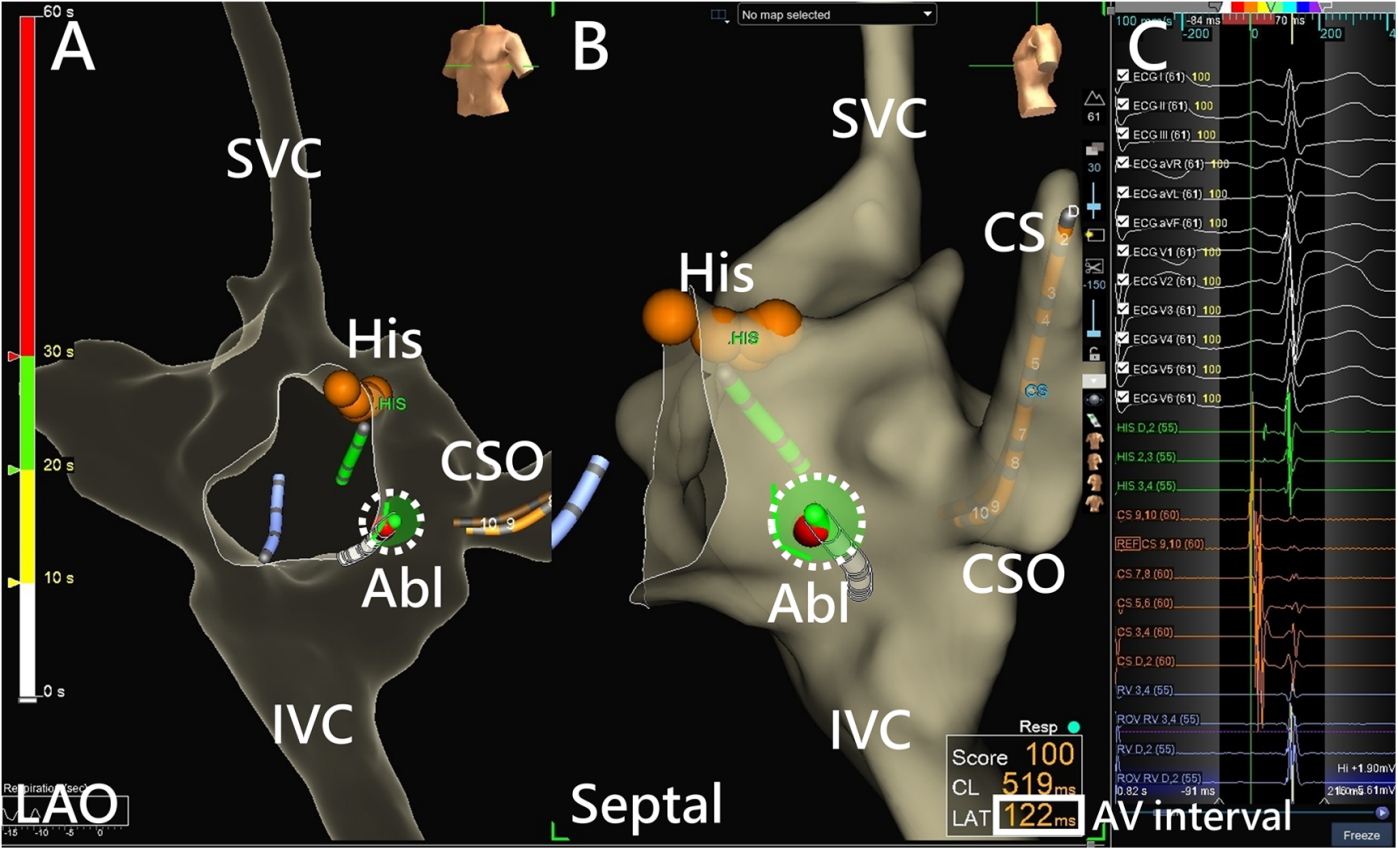
AVNRT ablation using a 3D EAM system. **(A)** RF ablation in the low Koch region on an LAO projection using a 3D EAM system, with the red dots indicating the His cloud. **(B)** A clearer spatial relationship between the ablation catheter and the Koch triangle is shown using a septal projection. Catheter contact can be assessed from local electrograms or the projection point area of the ablation catheter tip (dotted circle). **(C)** Monitoring of the real-time AV interval (bolded square) monitoring is achieved using the adequate window of the 3D EAM system during ablation. Abl, ablation catheter; AV interval, atrioventricular interval; CS, coronary sinus; CSO, coronary sinus ostium; His, bundle of His; IVC, inferior vena cava; LAO, left anterior oblique; SVC, superior vena cava.

A 5 Fr or 7 Fr nonirrigated radiofrequency ablation catheter (Ablaze Fantasista, Japan Lifeline, Tokyo, Japan; Livewire, Abbott, MN, USA) was used. In 3D geometry (Figure 1), mapping of Koch's triangle was established using the previously marked locations of His bundle and the coronary sinus (CS) ostium base on the CS catheter. The ideal location of ablation was below the CS roof with an AV ratio of 1: 2–1: 5 and a “hump-and-spike” atrial electrogram. At eligible sites, RF energy applications (maximum, 50°C, 50 W) were delivered. If an accelerated junctional rhythm was observed, a 60-second RF ablation would be administered under atrial overdrive pacing. If there were more than two consecutive accelerated junctional beats or AH prolongation, the operator would temporarily stop ablation using a foot pedal control. Ablation would then be immediately resumed after confirming intact antegrade AV node conduction. The real-time AV interval was also monitored by the 3D mapping system. After a total of 60 s of RF applications, the presence and function of the slow pathway were evaluated. A booster RF application of 30–60 s was given if the slow pathway was eliminated or if there was only one AV nodal echo beat. A repeated EPS with isoproterenol infusion was performed after ablation.

### 2.4 Cryoablation

A 6 mm or 8 mm tip cryoablation catheter (Freezor Xtra or Freezor Max, Medtronic, USA) was used. Once the location of the slow AV nodal pathway was established by an anatomic approach and ablation response with 3D EAM guidance, cryotherapy was applied during sinus rhythm or during AVNRT. For those ablated during sinus rhythm, cryo-mapping was first performed by applying cryothermal energy to −80°C for 1 min. During cryo-mapping, repeated atrial extrastimulation tests were performed to document a change in slow pathway conduction of the AV node and lack of AVNRT inducibility after 20 s of cryoablation. For those ablated during AVNRT, cryo-mapping was performed by applying cryothermal energy to −80°C for 20 s. During the period, termination of AVNRT due to slow pathway conduction block within 20 s was considered a slow pathway location. If AVNRT took longer than 20 s to stop, an alternative site would be chosen. Whether during sinus rhythm or AVNRT, cryoablation was administered at −80°C for a total of 4 min at the slow pathway location. In the same position, a freeze-thaw-freeze protocol was applied, with an initial 4-minute ablation followed by thawing and a second 4-minute freeze. Four two-minute subsequent cryoablation lesions were placed by surrounding the initial site. After cryoablation, a repeated EPS with isoproterenol infusion was performed.

### 2.5 Post-procedure monitoring and follow-up

All patients were hospitalized overnight after the procedure. A 12-lead ECG and an echocardiography were performed the day after the procedure. These patients were followed up at 1, 6, 12 and 24 months after the procedure. During follow-up, symptoms of recurrent AVNRT and ECG were evaluated. Recurrence was defined as the recurrence of clinical symptoms with documented abnormal heart rate or documented AVNRT ECG.

### 2.6 Study outcomes

The main outcome of interest for this study was AVNRT-free survival, which was defined as the acute success of catheter ablation and the absence of AVNRT recurrence during the follow-up period. The acute success of AVNRT ablation is defined as noninducible AVNRT with no evidence of AH or VA jump (slow pathway elimination) or a maximum of one AV nodal echo beat during isoproterenol infusion (slow pathway modification). The collected data, including procedural complications, procedure time, fluoroscopy time, location of the successful site, ablation lesions, ablation time and slow pathway treatment result, were analyzed and compared. Furthermore, we defined prolonged procedure time or not by using the third quartile of procedure time as the cut-off point.

### 2.7 Statistics

The Statistical Package for Social Sciences statistical software (SPSS, Version 22 for Windows) was used for statistical analyzes. Continuous variables are presented as medians with interquartile ranges (IQR), and nominal variables are presented as frequencies with associated percentages. For two-group comparisons, we used the Mann-Whitney *U*-test for continuous variables and the chi-square test (or Fisher's exact test) for nominal variables. Kaplan-Meier analysis was used to determine the freedom from AVNRT. The log-rank test was performed to test the difference between two groups. We used univariate and multivariate logistic regression models to evaluate the risk factor for a prolonged procedure time. Odds ratios (OR) and 95% confidence intervals (CI) were calculated for each model. The scatter plot between procedure time and the time sequence of the ablation between the two groups was analyzed using Spearman's rho correlation. A *P* value less than 0.05 was considered significant and all tests were 2-tailed.

## 3 Results

### 3.1 Baseline characteristics

Among the 95 patients included in this study, the median age was 13.5 years and 49 were female (51.6%). Four patients with congenital or structural heart diseases were enrolled (1 in the RF group and 3 in the Cryo group, *p* = 0.061). Two patients in the Cryo group had previously received an ablative procedure for AVNRT, while none in the RF group had. Three cases in the Cryo group had undergone initial RF ablation during the same procedure; one due to failed ablation over the lower Koch region in a case of complex congenital heart disease, and two because of transient complete AV block during the procedure, which led to crossover to cryoablation. Other demographic data was comparable between the groups, including body weight (Table 1). While the overall age distribution between the two groups did not show a significant difference, a subgroup analysis (Supplementary Table S1) revealed a significantly higher proportion of patients receiving cryoablation in the youngest quartile (≤11.5 years).

**Table 1:** Baseline characteristics.

	Total (*n* = 95)		RF group (*n* = 69)		Cryo group (*n* = 26)		*P* value
Age (year)	13.5	(11.5–15.9)	13.6	(12.3–15.7)	11.9	(9.8–16.0)	0.129
Gender—female	49	(51.6%)	32	(46.4%)	17	(65.4%)	0.098
Body weight (kg)	49.0	(38.3–62.0)	51.0	(38.6–62.5)	41.6	(34.4–56.3)	0.121
Body surface area (m^2^)	1.46	(1.25–1.72)	1.50	(1.28–1.72)	1.32	(1.18–1.58)	0.095
Structural/congenital heart disease	4	(4.2%)	1	(1.5%)	3	(11.5%)	0.061
Previous AVNRT ablation	2	(2.1%)	0	(0%)	2	(7.7%)	0.073
Antiarrhythmic drug	37	(39.0%)	25	(36.2%)	12	(46.2%)	0.377
Clinically documented tachycardia	76	(80.0%)	54	(78.3%)	22	(84.6%)	0.490

AVNRT, atrioventricular nodal reentry tachycardia.

### 3.2 Electrophysiological characteristics

During EPS, most of the patients had inducible AVNRT (93.7%), with typical AVNRT being the predominant subtype (79.0%). A lower percentage of patients presented with atypical AVNRT (14.7%). A single slow pathway was revealed in 83.2% of the patients, with the majority of antegrade slow pathways (65.3%). A lower common pathway block was observed in 11 patients (11.6%). SSPC was found in 22 patients (23.2%). No significant differences in these electrophysiological characteristics were observed between the groups. The most frequently induced coexisting arrhythmia was atrial fibrillation or atrial flutter, mostly non-sustained (Table 2).

**Table 2:** Electrophysiology characteristics.

	Total (*n* = 95)		RF group (*n* = 69)		Cryo group (*n* = 26)		*P* value
AVNRT type							0.099
Typical	75	(79.0%)	56	(81.2%)	19	(73.1%)	
Atypical	14	(14.7%)	11	(15.9%)	3	(11.5%)	
DAVN	6	(6.3%)	2	(2.9%)	4	(15.4%)	
Number of slow pathways							0.671
Single	79	(83.2%)	56	(81.2%)	23	(88.5%)	
Double	15	(15.8%)	12	(17.4%)	3	(11.5%)	
Triple	1	(1.0%)	1	(1.4%)	0	(0%)	
Slow pathway direction							0.447
Antegrade	62	(65.3%)	43	(62.3%)	19	(73.1%)	
Retrograde	3	(3.2%)	2	(2.9%)	1	(3.8%)	
Bidirectional	30	(31.6%)	24	(34.8%)	6	(23.1%)	
Lower common pathway block	11	(11.6%)	8	(11.6%)	3	(11.5%)	>0.99
SSPC	22	(23.2%)	15	(21.7%)	7	(26.9%)	0.593
Other tachycardia	30	(31.6%)	25	(36.2%)	5	(19.2%)	0.112
AF or AFL	23	(76.7%)	21	(84.0%)	2	(40.0%)	0.068
Atrial tachycardia	2	(6.7%)	1	(4.0%)	1	(20.0%)	0.310
Accessory pathway	5	(16.7%)	4	(16.0%)	1	(20.0%)	>0.99
Ventricular tachycardia	1	(3.3%)	0	(0%)	1	(20.0%)	0.167

AF, atrial fibrillation; AFL, atrial flutter; AV, atrioventricular; AVNRT, atrioventricular nodal reentry tachycardia; DAVN, dual atrioventricular nodes; SSPC, sustained slow pathway conduction.

### 3.3 Procedure and clinical outcomes

The 3D EAM system was used in all cases. More patients in the RF group received a nonfluoroscopic procedure (89.9% vs. 50%, *p* < 0.001). For those who received fluoroscopy during the procedure, the median fluoroscopic time was 6.9 min in the RF group and 6.8 min in the Cryo group (*p* = 0.438). The procedure time was significantly shorter in the RF group (111 min vs. 153.5 min, *p* = 0.005). The ablation time was also shorter in the RF group (4.7 min vs. 24 min, *p* < 0.001) (Table 3).

**Table 3:** Procedural and clinical outcomes.

	Total (*n* = 95)		RF group (*n* = 69)		Cryo group (*n* = 26)		*P* value
Slow pathway location							<0.001
Low Koch	67	(70.5%)	56	(81.2%)	11	(42.3%)	
Non-lower Koch	28	(29.5%)	13	(18.8%)	15	(57.7%)	
Acute success	92	(96.8%)	68	(98.6%)	24	(92.3%)	0.181
Slow pathway treatment result (*n* = 92)							0.787
Elimination	40	(43.5%)	29	(42.7%)	11	(45.8%)	
Modification	52	(56.5%)	39	(57.3%)	13	(54.2%)	
Residual slow pathway after ablation (*n* = 91)							0.429
1 AV nodal echo without ISO	19	(20.9%)	16	(23.5%)	3	(13.0%)	
1 AV nodal echo only with ISO	22	(24.2%)	17	(25.0%)	5	(21.7%)	
No echo, but jump or SSPC ± ISO	9	(9.9%)	5	(7.4%)	4	(17.4%)	
No slow pathway conduction ± ISO	41	(45.1%)	30	(44.1%)	11	(47.8%)	
Ablation lesions	22	(9–39)	28	(12.5–45)	8.5	(5–18.25)	<0.001
Ablation time (min)	6.6	(3.8–17.8)	4.7	(3.3–7.4)	24	(17.5–36.9)	<0.001
Procedure time (min)	125.0	(96.0–167.0)	111.0	(93.5–147.5)	153.5	(106.8–187.8)	0.005
Nonfluoroscopic procedure	75	(79.0%)	62	(89.9%)	13	(50.0%)	<0.001
Fluoroscopic time (min)	6.9	(2.4–20.1)	6.9	(2.8–29.8)	6.8	(1.4–16.7)	0.438
Minor complications	13	(13.7%)	10	(14.5%)	3	(11.5%)	>0.99
Transient AV block	9	(9.5%)	6	(8.7%)	3	(11.5%)	0.702
Follow-up duration (mon)	21.6	(12.9–30.9)	20.6	(13.3–28.4)	25.2	(7.4–48.8)	0.153
Recurrence	2	(2.2%)	2	(2.9%)	0	(0%)	>0.99

AV, atrioventricular; ISO, isoproterenol; SSPC, sustained slow pathway conduction.

The acute success rate was 98.6% in the RF group and 92.3% in the Cryo group (*p* = 0.181). The 3 failed cases (1 in the RF group and 2 in the Cryo group) were all received ablation on the low Koch, middle Koch, and coronary sinus ostium.

**Figure 2:**
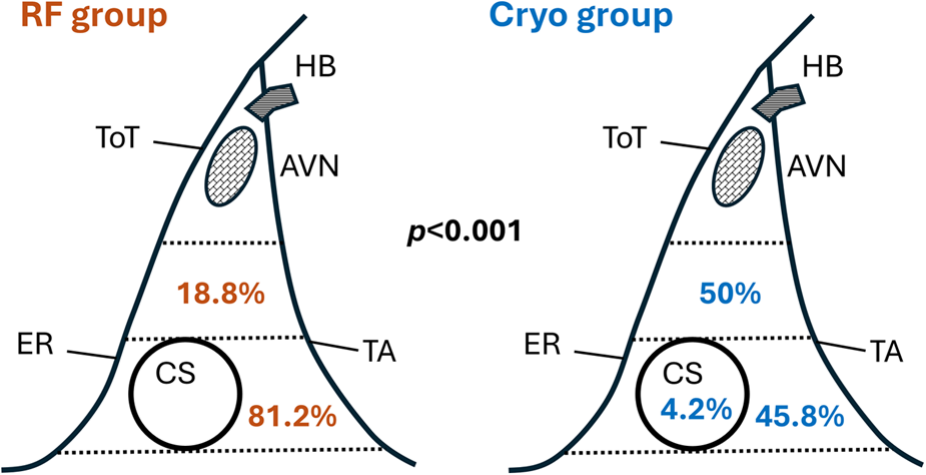
Distribution of slow pathway ablation sites for RF and cryo groups. AVN, atrioventricular node; CS, coronary sinus; ER, Eustachian ridge; HB, His bundle; TA, tricuspid annulus; ToT, tendon of Todaro.

In the RF group, the site of success for slow pathway ablation was predominantly in the low Koch region (81.2%). However, in the Cryo group, 50% were located in the middle Koch region and 45.8% in the low Koch region (*p* < 0.001) (Figure 2).

Modification of the slow pathway was slightly more prevalent (56.8%) compared to elimination of the slow pathway (43.2%), with a similar distribution between the groups. EPS after catheter ablation showed that 17.3% of the patients had residual single atrioventricular node (AVN) echo without isoproterenol infusion, 28.4% had residual single AVN echo only with isoproterenol infusion, 9.9% had no echo beat under physiological conditions but ongoing evidence of jump in AH/VA interval or SSPC, and 44.4% had no evidence of slow pathway conduction with or without isoproterenol infusion. The distribution of residual slow pathway conduction was similar between the groups.

None of the patients had a complete or high-grade AV block that warranted pacemaker implantation. All documented complications were considered minor, including 9 patients with transient AV block that recovered within the procedure, one with Mobitz type 1 AV block, and 2 with right bundle branch block that recovered during the follow-up period. No significant differences in complication rates were found between the groups (14.5% vs. 11.5%).

**Figure 3:**
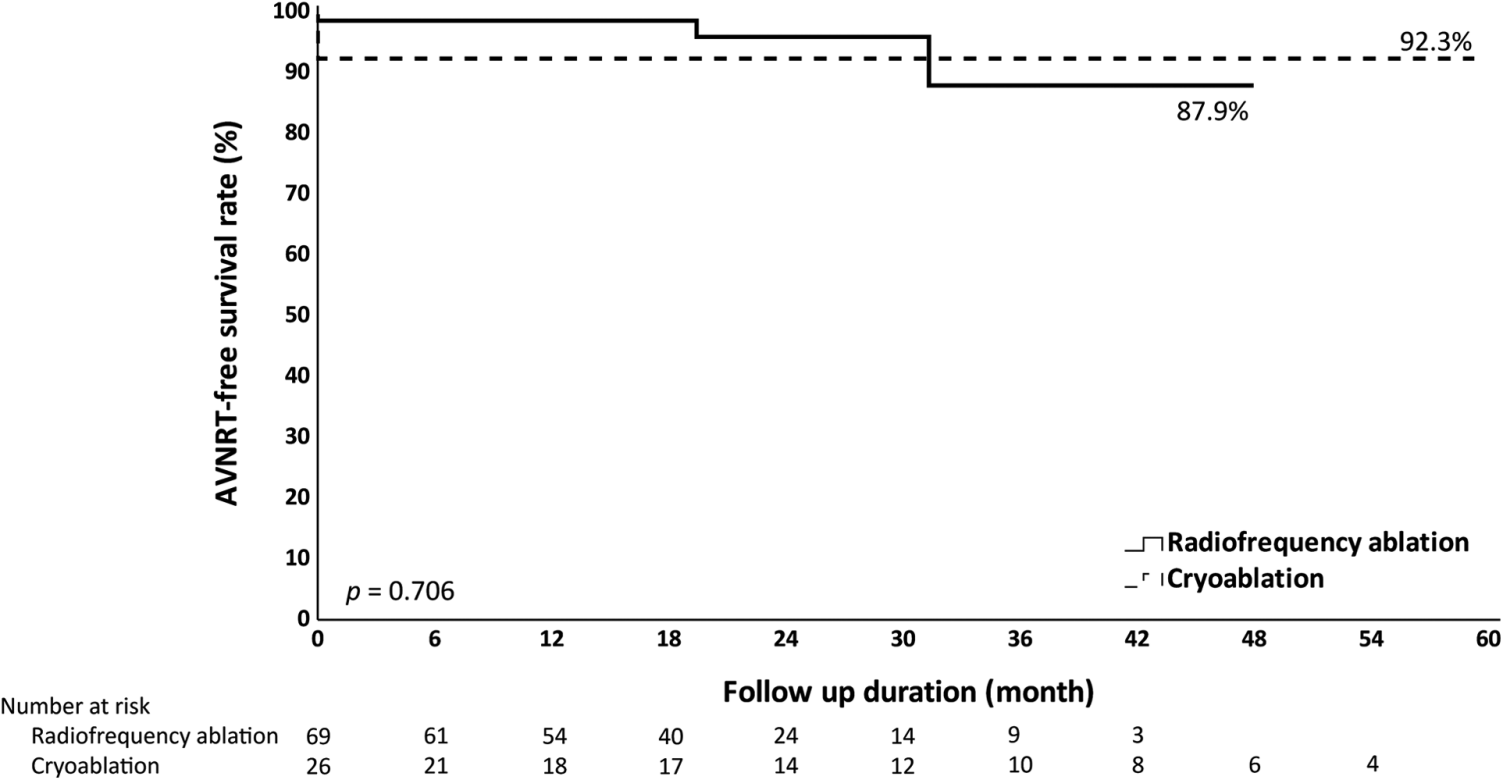
Kaplan-Meier analysis of AVNRT-free survival. Kaplan-Meier analysis does not show significant differences in AVNRT-free survival when comparing radiofrequency ablation and cryoablation.

Both groups had a similar median follow-up duration, which was 20.6 months in the RF group and 25.2 months in the Cryo group. During the follow-up period, there was no difference in AVNRT recurrence between the groups, that two patients in the RF group and none in the Cryo group had documented AVNRT recurrence (2.9% vs. 0%). Furthermore, there were no differences in AVNRT-free survival by Kaplan-Meier analysis (Figure 3).

### 3.4 Risk factors for prolonged procedure time

In the univariate analysis (Supplementary Table S2), cryoablation, the occurrence of a transient AV block, using fluoroscopy, and ablation outside the lower Koch triangle were significantly associated with a prolonged procedure time (longer than the third quartile, 167 min). In the multivariate analysis (Table 4), three separate models were examined. Model 1, which combined ablation energy type and fluoroscopy use, confirmed that cryoablation was significantly associated with longer procedure time. In Model 2, which combined ablation energy type and transient AV block, cryoablation remained significantly associated with longer procedure time. However, in Model 3, which combined ablation energy type and ablation site, ablation outside the lower Koch triangle was significantly associated with longer procedure time, while the type of ablation energy did not show a significant difference in this model.

**Table 4:** Multivariate logistic regression analyses for procedure time (≧167 min).

	Model 1			Model 2			Model 3		
	OR	(95% CI)	*P* value	OR	(95% CI)	*P* value	OR	(95% CI)	*P* value
Ablation energy type									
RF ablation	ref.			ref.			ref.		
Cryoablation	3.94	(1.33–11.68)	0.013	4.91	(1.77–13.60)	0.002	2.90	(0.98–8.58)	0.055
Nonfluoroscopic procedure	0.61	(0.19–2.01)	0.421						
Transient AV block				4.44	(0.99–19.97)	0.052			
Slow pathway location									
Lower Koch							ref.		
Non-lower Koch							4.73	(1.63–13.69)	0.004

AV, atrioventricular; RF, radiofrequency; ref., reference.

### 3.5 Learning curve in different ablation energy groups

**Figure 4:**
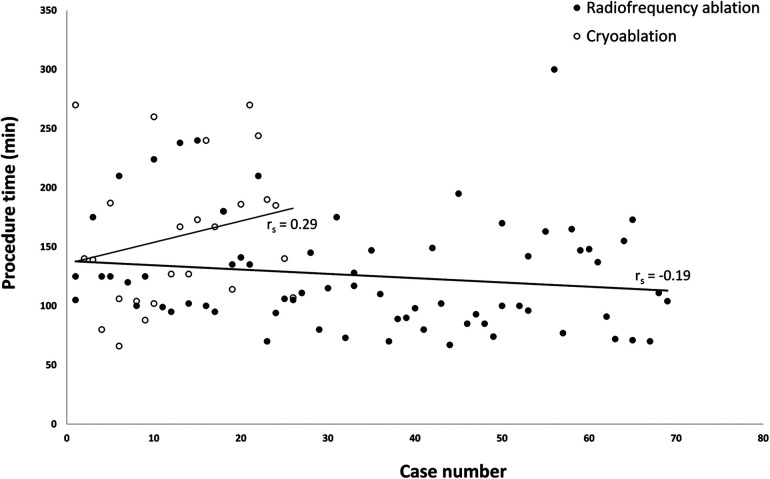
Scatter plot of procedure time versus case rank by the time of operation. The scatter plot does not show significant differences in procedure time with an increasing number of procedures in both groups (Spearman's rho *p* > 0.05). However, a trend of decreased procedure time was observed in the radiofrequency ablation group and a trend of increased procedure time in the cryoablation group.

We compared procedure time between the first half and the second half of cases and also in the subgroup of RF ablation and cryoablation. Only RF ablation showed a significant reduction in procedure time for the second half, while no significant differences were observed in the overall group or cryoablation (Supplementary Table S3). There are trends of increasing procedure time in cryoablation and decreasing procedure time in RF ablation, but without reaching statistical significance (Figure 4). The achievement of nonfluoroscopic ablation significantly increased in the recent 50% of cases in the overall group (62.5% to 95.7%) (Supplementary Table S4).

## 4 Discussion

This study compares RF ablation and cryoablation for the treatment of pediatric AVNRT in the context of 3D EAM guidance. The results provide insight into the comparative outcomes of both procedures with high acute success rates (98.6% for RF ablation and 92.3% for cryoablation) and low recurrence rates (2.9% for RF ablation and 0% for cryoablation). No incidence of permanent AV block or other major complications was observed in either ablative modality. Also, RF ablation is associated with a shorter procedure time (111 min vs. 153.5 min).

Several published studies compare the clinical results of RF ablation and cryoablation for the treatment of pediatric AVNRT. Collins et al. (12) reported similar clinical outcomes between RF ablation and cryoablation using a 4 mm cryoablation catheter, with respect to short-term efficacy and safety. Unlike our data, a trend of a higher recurrence rate was found in cryoablation (2% for RF ablation vs. 8% for cryoablation, *p* = 0.19). Papagiannis et al. (13) also reported the comparative effectiveness and safety of the two techniques using a 6 mm cryoablation catheter and a higher rate of recurrence in cryoablation after long follow-up periods (10% for RF ablation vs. 27.7% for cryoablation, *p* = 0.222). Backhoff et al. (3) reported long-term outcomes of children who underwent successful AVNRT ablation (excluding cases of ablation failure and permanent AV block immediately after RF ablation) with the EAM system used in all cases. A similar prevalence of AVNRT recurrence was observed between RF ablation and cryoablation. Recently, Shah et al. (14) reported a nationwide registry study of over 2,000 pediatric patients comparing RF ablation and cryoablation for AVNRT. The study found similar acute success rates, no cases requiring permanent pacing, and repeat ablation more common in the cryoablation group. In our study, the lower incidence of AVNRT recurrence in the Cryo group could potentially be explained by the detailed cryomapping protocol.

The decision of whether RF ablation or cryoablation should be used to treat pediatric patients with AVNRT remains somewhat controversial. Although a rare complication, complete AV block is the main concern for RF ablation, which often requires a lifelong pacemaker and can have a prolonged impact on quality of life and overall health, especially in children. On the other hand, complete AV block has never been reported with cryoablation in previous studies. In the current era of the 3D EAM system, it is crucial to understand whether the use of RF ablation in pediatric patients with AVNRT, aided by the advanced mapping system, results in a reduced incidence of complete AV block. In a recent prospective multicenter study, 1,007 adult patients with AVNRT received RF ablation without fluoroscopy and none had AV block (15). The result suggests that nonfluoroscopic RF ablation of the slow pathway can be performed safely in adults. In our study, none of the pediatric patients with AVNRT who underwent RF ablation developed complete AV block. Our strategies to prevent AV block in pediatric AVNRT include (1) marking the position of His bundle in the 3D geometry, (2) continuous monitoring on the quantitative AV interval via the 3D EAM system, (3) pedal control for the initiation and termination of RF applications by the operator, and (4) a smaller local A/V ratio ranged from 1:4 to 1:5 when applications of the middle third Koch triangle needed.

Regarding procedural efficiency, our study highlights that RF ablation in the era of 3D EAM is generally associated with a shorter procedure time in pediatric patients with AVNRT. This trend is generally consistent in our multivariate analyses. This finding aligns with the results of two meta-analyses conducted in adult populations (8, 9) and one of the pediatric cohort studies (12) without the guidance of 3D EAM. In our experience, the difference in procedural efficiency between the two modalities studied may be attributed to the additional time required to perform multiple cryoablations, such as mapping and a longer cryoablation time for the successful site.

The comparison of procedure time between the first half and the last half of the cases indicates a significant reduction in the procedure time for RF ablation with increasing experience. This suggests that RF ablation strongly depends on a learning curve effect, where accumulated experience and familiarity with the technique lead to more efficient procedures. On the contrary, cryoablation did not show a similar trend and exhibited a tendency for longer procedure time even with more experience.

Catheter ablation guided by fluoroscopy, whether through RF ablation or cryoablation, is a well-established technique for treating AVNRT in children. However, the exposure to ionizing radiation in EP procedures is not negligible for both patients and laboratory staff. The NO-PARTY trial demonstrated a significant 96% reduction in the estimated risks of cancer incidence and mortality when using a nonfluoroscopic approach compared to the conventional method, without compromising procedural safety and efficacy (16). Given the longer life expectancy and the ongoing development of organ systems in children, there is a greater likelihood of expressing the stochastic effects of radiation. 3D EAM-guided RF ablation or cryoablation to treat patients with SVT have been demonstrated to significantly reduce the reliance on fluoroscopy (17), further advancing the feasibility of non-fluoroscopic procedures. To date, several studies have been published reporting the outcomes of nonfluoroscopic RF ablation or cryoablation in the treatment of pediatric AVNRT (5, 18–24). In our study, nearly 80% of AVNRT ablation procedures were successfully performed using a nonfluoroscopic method, with this proportion rising to 95.7% in the latter half of the cases. This finding highlights the potential for nonfluoroscopic techniques to become the standard of care. To our knowledge, our study contains the largest published cohort of pediatric patients with AVNRT who have undergone nonfluoroscopic radiofrequency ablation, further validating the feasibility and effectiveness of this approach.

Based on our findings, RF ablation may be the preferred choice in most cases when ablating over the lower Koch triangle, considering its generally shorter procedure time and comparable safety profile. Cryoablation remains a valuable option, especially for younger children or when the ablation site is located outside the lower Koch triangle. Its excellent safety profile, particularly in minimizing the risk of AV block, makes it an appropriate choice in anatomically complex or high-risk scenarios.

## 5 Study limitations

First, this study is a retrospective observational study with a relatively small patient population, which may lead to an underestimation of event rates, such as complications or recurrence. However, the *post hoc* power analysis based on the procedure time showed power of 0.78, indicating a reasonable ability to detect a medium effect size (Cohen's d = 0.65). Second, more patients in the RF group received a nonfluoroscopic procedure with respect to the preference of the operators. This could potentially result in bias that affects the study results. Another limitation is the potential impact of operator variability and center-specific protocols on the outcomes, despite the fact that the protocols between the two centers were largely similar. Finally, the substantial difference in the sizes of the RF ablation and cryoablation compared groups (69 vs. 26 patients) imposes limitations on the validity of our analysis.

## 6 Conclusions

Under the guidance of the 3D EAM system, both cryoablation and RF ablation are considered effective and safe in pediatric patients with AVNRT. Moreover, RF ablation is more efficient and is associated with a significantly shorter procedure time after increasing experience.

## Data Availability

The original contributions presented in the study are included in the article/Supplementary Material, further inquiries can be directed to the corresponding author.
