# Supplementary material for: Radiofrequency ablation vs. cryoablation for pediatric atrioventricular nodal reentrant tachycardia in the era of three-dimensional electroanatomical mapping
Source: Front Cardiovasc Med. 2025 Jan 30;12:1527768. doi: 10.3389/fcvm.2025.1527768 (PMC11821964; doi:10.3389/fcvm.2025.1527768)
Supplement: Supplementary file 1 [file Table1.docx]

Supplementary Material

# Supplementary Tables

| **Supplementary Table S1**. Demographic and procedural data by age quartile. | | | | | |
| --- | --- | --- | --- | --- | --- |
|  | Age quartile 1 (n=24) | | Age quartiles 2–4 (n=71) | | *P* value |
|  | Median | IQR | Median | IQR |  |
| Age (year) | 9.2 | (7.2-10.5) | 14.7 | (13.1-16.3) | <0.001 |
| Gender - female, n (%) | 12 | (50.0%) | 37 | (52.1%) | 0.858 |
| Body weight (kg) | 32.8 | (22.1-39) | 55.3 | (44.8-65) | <0.001 |
| Structural/congenital heart disease, n (%) | 4 | (16.7%) | 0 | (0%) | 0.003 |
| Previous AVNRT ablation, n (%) | 0 | (0%) | 2 | (2.8%) | >0.99 |
| Antiarrhythmic drug, n (%) | 11 | (45.8%) | 26 | (36.6%) | 0.424 |
| AVNRT type, n (%) |  |  |  |  | 0.247 |
| Typical | 19 | (79.2%) | 56 | (78.9%) |  |
| Atypical | 2 | (8.3%) | 12 | (16.9%) |  |
| DAVN | 3 | (12.5%) | 3 | (4.2%) |  |
| Other tachycardia, n (%) | 9 | (37.5%) | 21 | (29.6%) | 0.470 |
| AF or AFL | 7 | (77.8%) | 16 | (76.2%) | >0.99 |
| Atrial tachycardia | 0 | (0%) | 2 | (9.5%) | >0.99 |
| Accessory pathway | 2 | (22.2%) | 3 | (14.3%) | 0.622 |
| Ventricular tachycardia | 0 | (0%) | 1 | (4.8%) | >0.99 |
| Type of ablation energy, n (%) |  |  |  |  | 0.004 |
| Radiofrequency ablation | 12 | (50.0%) | 57 | (80.3%) |  |
| Cryoablation | 12 | (50.0%) | 14 | (19.7%) |  |
| Slow pathway location, n (%) |  |  |  |  | 0.631 |
| Lower Koch | 16 | (66.7%) | 51 | (71.8%) |  |
| Non-lower Koch | 8 | (33.3%) | 20 | (28.2%) |  |
| Acute success, n (%) | 23 | (95.8%) | 69 | (97.2%) | >0.99 |
| Slow pathway treatment result, n (%) (n=92) |  |  |  |  | >0.99 |
| Elimination | 10 | (43.5%) | 30 | (43.5%) |  |
| Modification | 13 | (56.5%) | 39 | (56.5%) |  |
| Procedure time (min) | 127.5 | (94.8-178.8) | 120.0 | (96-163) | 0.601 |
| Fluoroscopic guidance, n (%) |  |  |  |  | 0.259 |
| Nonfluoroscopic | 17 | (70.8%) | 58 | (81.7%) |  |
| Fluoroscopic | 7 | (29.2%) | 13 | (18.3%) |  |
| Fluoroscopic time (min) | 6.8 | (0.1-15.2) | 6.9 | (2.5-20.9) | 0.588 |
| Minor complication, n (%) | 2 | (8.3%) | 11 | (15.5%) | 0.505 |
| Transient AV block, n (%) | 2 | (8.3%) | 7 | (9.9%) | >0.99 |
| Follow-up duration (mon) | 19.1 | (14.1-29.2) | 22.7 | (13.3-33.5) | 0.566 |
| Recurrence, n (%) | 0 | (0%) | 2 | (2.9%) | >0.99 |
| AF = atrial fibrillation; AFL = atrial flutter; AV = atrioventricular; AVNRT = atrioventricular nodal reentry tachycardia; DAVN = dual atrioventricular nodes. | | | | | |

| **Supplementary Table S2**. Univariate logistic regression analyses for procedure time (≧167 min). | | | | |
| --- | --- | --- | --- | --- |
|  | OR | (95% CI) | | *P* value |
| Ablation energy type |  |  |  |  |
| RF ablation | ref. |  |  |  |
| Cryoablation | 4.75 | (1.77-12.77) | | 0.002 |
| Age (year) | 0.93 | (0.81-1.07) | | 0.311 |
| Body weight (kg) | 1.01 | (0.98-1.03) | | 0.502 |
| Antiarrhythmic drug | 0.84 | (0.33-2.17) | | 0.725 |
| AVNRT type |  |  |  |  |
| Typical | ref. |  |  |  |
| Atypical | 2.56 | (0.78-8.40) | | 0.121 |
| DAVN | 1.71 | (0.29-10.13) | | 0.557 |
| Other tachycardia | 1.03 | (0.39-2.74) | | 0.958 |
| Slow pathway location |  |  |  |  |
| Lower Koch | ref. |  |  |  |
| Non-lower Koch | 6.58 | (2.42-17.91) | | <0.001 |
| Slow pathway treatment result (n=92) |  |  |  |  |
| Elimination | ref. |  |  |  |
| Modification | 1.91 | (0.69-5.26) | | 0.210 |
| Residual slow pathway after ablation (n=91) |  |  | |  |
| 1 AV nodal echo without ISO | ref. |  | |  |
| 1 AV nodal echo only with ISO | 0.64 | (0.16-2.56) | | 0.525 |
| No echo, but jump or SSPC ±ISO | 0.62 | (0.10-3.92) | | 0.610 |
| No slow pathway conduction ±ISO | 0.53 | (0.15-1.81) | | 0.308 |
| Nonfluoroscopic procedure | 0.33 | (0.12-0.94) | | 0.037 |
| Minor complications | 2.84 | (0.85-9.48) | | 0.089 |
| Transient AV block | 4.12 | (1.01-16.84) | | 0.048 |
| AV = atrioventricular; AVNRT = atrioventricular nodal reentry tachycardia; DAVN = dual atrioventricular nodes; RF = radiofrequency. | | | | |

| **Supplementary Table S3**. Learning curve on procedural time (min). | | | | | | | |
| --- | --- | --- | --- | --- | --- | --- | --- |
|  | First 50% cases | | | Late 50% cases | | | *P* value |
|  | n | Median | IQR | n | Median | IQR |  |
| Total | 48 | 125 | (100.5-162) | 47 | 114 | (90-170) | 0.685 |
| RF ablation | 35 | 125 | (100-147) | 34 | 100 | (83.75-148.25) | 0.049 |
| Cryoablation | 13 | 127 | (95-177) | 13 | 180 | (133.5-215) | 0.055 |
| RF = radiofrequency. | | | | | | | |

| **Supplementary Table S4**. Fluoroscopic vs. non-fluoroscopic procedures | | | | | |  |
| --- | --- | --- | --- | --- | --- | --- |
|  | First 50% cases | | Late 50% cases | | *P* value | |
|  | n | (%) | n | (%) |  |  |
| Fluoroscopic | 18 | (37.5%) | 2 | (4.3%) | <0.001 | |
| Nonfluoroscopic | 30 | (62.5%) | 45 | (95.7%) |  |  |
|  | | | | | |  |
